# Interactions between Glutathione *S-*Transferase P1, Tumor Necrosis Factor, and Traffic-Related Air Pollution for Development of Childhood Allergic Disease

**DOI:** 10.1289/ehp.11117

**Published:** 2008-03-25

**Authors:** Erik Melén, Fredrik Nyberg, Cecilia M. Lindgren, Niklas Berglind, Marco Zucchelli, Emma Nordling, Jenny Hallberg, Magnus Svartengren, Ralf Morgenstern, Juha Kere, Tom Bellander, Magnus Wickman, Göran Pershagen

**Affiliations:** 1 Institute of Environmental Medicine, Karolinska Institutet, Stockholm, Sweden; 2 Astrid Lindgren Children’s Hospital, Karolinska University Hospital, Stockholm, Sweden; 3 AstraZeneca R&D Mölndal, Mölndal, Sweden; 4 Department of Biosciences at Novum, Karolinska Institutet, Stockholm, Sweden; 5 Wellcome Trust Centre for Human Genetics, University of Oxford, Oxford, United Kingdom; 6 Department of Occupational and Environmental Health, Stockholm County Council, Stockholm, Sweden; 7 BEA Bioinformatics Core Facility at Novum, Karolinska Institutet, Stockholm, Sweden; 8 Department of Public Health Sciences, Karolinska Institutet, Stockholm, Sweden; 9 Sachs Children’s Hospital, Stockholm, Sweden

**Keywords:** ADRB2, air pollution, allergy, asthma, genetics, GSTP1, interaction, nitrogen oxides, polymorphism, TNF

## Abstract

**Background:**

Air pollutants may induce airway inflammation and sensitization due to generation of reactive oxygen species. The genetic background to these mechanisms could be important effect modifiers.

**Objective:**

Our goal was to assess interactions between exposure to air pollution and single nucleotide polymorphisms (SNPs) in the β*2-*adrenergic receptor (*ADRB2*), glutathione *S-*transferase P1 (*GSTP1*), and tumor necrosis factor (*TNF*) genes for development of childhood allergic disease.

**Methods:**

In a birth cohort originally of 4,089 children, we assessed air pollution from local traffic using nitrogen oxides (traffic NO_x_) as an indicator based on emission databases and dispersion modeling and estimated individual exposure through geocoding of home addresses. We measured peak expiratory flow rates and specific IgE for inhalant and food allergens at 4 years of age, and selected children with asthma symptoms up to 4 years of age (*n* = 542) and controls (*n* = 542) for genotyping.

**Results:**

Interaction effects on allergic sensitization were indicated between several *GSTP1* SNPs and traffic NO_x_ exposure during the first year of life (*p*_nominal_ < 0.001–0.06). Children with Ile105Val/Val105Val genotypes were at increased risk of sensitization to any allergen when exposed to elevated levels of traffic NO_x_ (for a difference between the 5th and 95th percentile of exposure: odds ratio = 2.4; 95% confidence interval, 1.0–5.3). In children with *TNF*-308 GA/AA genotypes, the *GSTP1*–NO_x_ interaction effect was even more pronounced. We observed no conclusive interaction effects for *ADRB2*.

**Conclusion:**

The effect of air pollution from traffic on childhood allergy appears to be modified by *GSTP1* and *TNF* variants, supporting a role of genes controlling the antioxidative system and inflammatory response in allergy.

Asthma and allergy are complex diseases in which numerous genetic and environmental factors play a role in the etiology. Air pollutants, including vehicle-related pollutants such as particles and nitrogen oxides (NO_x_), are known to induce airway inflammation and increase airway responsiveness (Gilmour et al. 2006; Grievink et al. 2000). It is plausible that genetic variants in the genes controlling the inflammatory and antioxidative systems may determine whether exposure to air pollutants will promote the development of allergic diseases. Identification of such gene–environment interactions may hold the key to understanding the great variability among individuals in responses to various environmental factors, among them environmental tobacco smoke, ozone, and diesel exhaust particles (London 2007).

The inflammatory response after exposure to air pollutants is maintained by the activation of proinflammatory molecules and may also induce lung damage due to generation of reactive oxygen species (MacNee 2001). Further, diesel exhaust particles may induce IgE responses directly by acting on B-cells and enhancing the production of cytokines that favor the development of an allergy-prone immune response (Sydbom et al. 2001).

Members of the glutathione *S*-transferase (GST) supergene family have been a particular focus of studies of gene–environment interactions, because they constitute an intracellular protective system against electrophiles and the formation of hazardous reactive oxygen species (Hayes and Strange 1995; McCunney 2005; Strange et al. 2001). Recent studies support the presence of gene–environment interaction, or effect modification, between exposure to air pollutants and glutathione *S-*transferase P1 (*GSTP1*) variants, especially the isoleucine (Ile)/valine (Val) polymorphism at amino acid position 105 with respect to childhood asthma and nasal allergic responses (Gilliland et al. 2004; Lee et al. 2004). Although GSTP1 is the GST enzyme most expressed in the lung (Fryer et al. 1986), deficiency of two other GST genes, *GSTM1* and *GSTT1*, has also been shown to influence the effect of passive smoking on the risk of childhood asthma and wheezing (Gilliland et al. 2002b; Kabesch et al. 2004). Other systems have also been implicated in interactions with air pollution. Interaction between exposure to ozone and tumor necrosis factor (*TNF superfamily, member 2*) polymorphisms, especially *TNF* G-308A, has been observed with regard to lung function and childhood wheezing (Li et al. 2006; Yang et al. 2005). Effect modification of exposure to passive smoking on the frequency of school absences due to respiratory illness has also been reported, supporting a role of TNF as a key player in relation to air pollutants (Wenten et al. 2005; Wu et al. 2007). Recently, the effect of passive smoking on asthma phenotypes in children was also reported to be modified by polymorphisms in the β*2-*adrenergic receptor (*ADRB2*) gene (Zhang et al. 2007), which supports previously observations in adults (Wang et al. 2001).

We have previously reported significant effects of exposure to traffic-related air pollutants during the first year life on respiratory symptoms, lung function, and sensitization up to 4 years of age in the Swedish birth cohort study BAMSE (the Children, Allergy, Milieu, Stockholm, Epidemiological Survey) (Nordling et al. 2008). The objective of the present study was to identify interactions between certain genetic variants and individual long-term exposure to air pollutants from traffic (evaluated as traffic-specific NO_x_) on the development of respiratory symptoms and allergic sensitization in children. We evaluated genes that have previously been identified as target genes for allergic diseases and with a potential for interaction with air pollutants in order to select suitable candidates from among genes that are involved in the inflammatory response and antioxidative pathway, or as airway receptors (Devalia et al. 1993; Forsberg et al. 2001; Holgate 1999; MacNee 2001; Wang et al. 2001). Thus, based on the considerations and prior data presented above, we selected the *TNF*, *ADRB2*, and *GSTP1* genes for analyses.

## Materials and Methods

### Study design

Between 1994 and 1996, 4,089 newborn infants were recruited in the BAMSE study, and questionnaire data on parental allergic diseases and residential characteristics were obtained (Melen et al. 2004b; Nordling et al. 2008). The catchment area included central and northwestern parts of Stockholm, that is, the municipalities Järfälla, Solna, and Sundbyberg and two inner-city districts, Vasastan and Norrmalm. Thus, both urban and suburban districts were represented. When their children were 1, 2, and 4 years of age, parents answered questionnaires on the children’s symptoms related to asthma and other allergic diseases. At approximately 4 years of age, 2,965 of the children participated in a clinical testing. At this visit, peak expiratory flow (PEF) tests were performed using the normal-range Ferraris peak flow meters (Ferraris Medical Ltd., Hertford, UK) (Hallberg et al. 2006). Each child recorded several PEF measurements, and we used the best PEF value for analysis. Seventeen children refused to do PEF measurements, and we excluded data from 349 children because of inability to perform acceptable tests (as judged by the test leader or inability to produce two reproducible PEF values within 15%), leaving 2,599 children with acceptable PEF-readings (88%). Blood samples were also drawn from 2,614 children.

**Figure 1:**
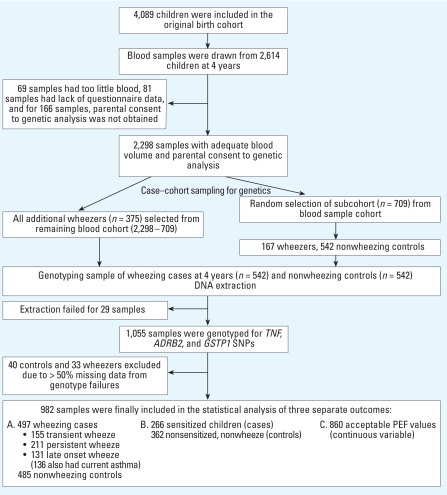
The BAMSE study design: selection of subjects for genotyping and exclusion of subjects following the genotyping procedure.

The study design for the genetics part of BAMSE has been presented elsewhere in detail (Melen et al. 2004a, 2004b, 2005). For the genetic analyses, 2,298 blood samples were available after exclusion of 69 samples because of too little blood, 81 samples because of lack of questionnaire data, and 166 samples because parental consent to genetic analysis of the sample was not obtained (Figure 1). From these 2,298 samples, for cost-efficiency reasons we selected a subsample for genetic analyses, using a case–cohort design. First, a randomly sampled subcohort of 709 children provided 542 nonwheezers as an unbiased control sample for analyses using this phenotype, as well as 167 wheezers. In addition, we selected all remaining wheezers among the 2,298, providing a further 375 wheezers, totaling 542 cases with the wheeze phenotype. We extracted DNA from all of the 1,084 selected samples. Extraction failed for 29 samples, leaving 1,055 samples for genotyping of *TNF*, *ADRB2*, and *GSTP1* single nucleotide polymorphisms (SNPs), but we subsequently excluded 40 controls and 33 wheezers because of > 50% missing data from genotype failures, indicating poor DNA quality. Thus, we included 982 children (497 wheezing cases, 485 controls, of whom 150 wheezers and 485 controls were from the subcohort) in the analyses.

The subcohort mirrors the full BAMSE cohort well in terms of sociodemographic factors, wheeze, and sensitization prevalence (Melen et al. 2004a). The subcohort obtained using the case–cohort design has the advantage of providing effect estimates for other outcomes than wheezing (e.g., sensitization and PEF) in a representative group of children that are guaranteed to be unbiased by the wheezing phenotype case–control selection. On the other hand, the sample size (*n* = 635) is smaller than the full genetics study sample (*n* = 982), and other phenotypes may be enriched among the wheezers, so if bias is not suspected to be an issue, analysis using the full sample size may instead be advantageous.

The study was approved by the ethical committee at Karolinska Institutet, and parental consent to genetic analyses was obtained for all participating children.

### Definitions of study phenotypes

We subdivided children with history of wheezing into three groups according to reported episodes during the early (3 months to 2 years) and recent (last 12 months at 4 years) age periods (Figure 1), using the following criteria (Melen et al. 2004b): *a*) transient wheezing: three or more episodes of wheezing between 3 months and 2 years of age, but no episode in the preceding 12 months at 4 years; *b*) persistent wheezing: one or more episodes of wheezing between 3 months and 2 years of age and one or more episodes in the preceding 12 months at 4 years; and *c*) late-onset wheezing: no episode of wheezing between 3 months and 2 years of age, but one or more episodes in the preceding 12 months at 4 years. We defined current asthma as reported physician-diagnosed asthma up to 4 years of age and one or more episodes of wheezing in the preceding 12 months at 4 years (nonwheezing children without asthma diagnosis at 4 years served as controls).

We measured sensitization to either inhalant or food allergens [serum IgE antibodies ≥ 0.35 kilounits of antibody per liter (kU_A_/L)] with Phadiatop (Phadia AB, Uppsala, Sweden): a mixture of cat, dog, horse, birch, timothy, mugwort, *Dermatophagoides pteronyssinus*, and *Cladosporium* allergens and fx5 (Phadia AB): a mixture of milk, egg white, soy bean, peanut, fish, and wheat allergens. Twenty-nine percent of the wheezing cases were sensitized, versus 25% of the controls [see Supplemental Material, Table 1 (http://www.ehponline.org/members/2008/11117/suppl.pdf)], in total providing 266 sensitized cases. To investigate whether the degree of sensitization might influence the results, we also used a higher cutoff for serum IgE antibodies (≥ 3.5 kU_A_/L) in some analyses. Of the 982 children with genotype information, 860 performed acceptable PEF tests (88%), and we used PEF as a continuous variable. In the subcohort of 635 children, 175 were sensitized to inhalant or food allergens (28%), and 557 (88%) had acceptable PEF measurements.

### Air pollution assessment

We based the assessment of exposure to locally emitted air pollution on a methodology developed to retrospectively estimate long-term source-specific exposure to air pollution in the study area, described in detail elsewhere (Nordling et al. 2008; Rosenlund et al. 2006). It entails geocoding of an individual’s address information and uses an emission inventory together with dispersion models to map outdoor levels of selected pollutants from selected emission sources over time at the relevant geographical locations. Thus, we used this methodology to assess the children’s individual exposure to traffic-related NO_x_ during their life but focusing on the first year of life.

We retrieved residential address information from the questionnaires at 2 months and 1 year of age and transformed this into geographical coordinates (geocoded) using standard geographical information systems computer software (MapInfo; Pitney Bowes MapInfo, Troy, NY, USA), in combination with an address database. Emission databases describing traffic-generated NO_x_ (traffic NO_x_) within the county were available for the years 1990 and 2000. We assessed the geographical distribution of air pollution in three different grid resolutions, applied to regional/countryside (500 × 500 m), urban (100 × 100 m), and inner-city (25 × 25 m) areas. In addition, because the air pollution levels in the city also depend on very local traffic conditions, we added a street canyon contribution for addresses in the most polluted street segments in the city center (3% of the addresses). We estimated the dispersion of pollutants from the sources with a dilution model based on the average annual distribution of wind speed, direction, and precipitation.

We calculated residential outdoor levels of traffic NO_x_ for each month at each address during each child’s first year of life (in the period 1994–1997) from the 1990 and 2000 values for that address, by interpolation between these two values assuming a linear change in air pollution levels between these years in each point. We verified close to linear trends from graphs of annual means for the years concerned.

In the previously reported main effect results for air pollution in the BAMSE cohort, Nordling et al. (2008) noted a high correlation between exposure to traffic NO_x_ and traffic particulate matter ≤ 10 μm in aerodynamic diameter (PM_10_) (estimated by similar methodology; *r* = 0.94), and we chose here to present only NO_x_ analyses as a proxy for air pollution exposure from traffic. Validation analyses showed good agreement (*r* = 0.74) between calculated traffic-related NO_2_ levels, based on our assessed annual traffic NO_x_ levels, and measured 1-month outdoor levels of ambient NO_2_ (Nordling et al. 2008).

### SNPs and genotyping

We extracted DNA from peripheral blood leukocytes using a standard nonenzymatic method or the Puregene kit (Gentra Systems, Minneapolis, MN, USA). We selected a number of registered SNPs [preferably with proven or suggested functional effects based on the existing literature or according to the National Center for Biotechnology Information (NCBI) database] in each of the *TNF*, *ADRB2*, and *GSTP1* genes from the NCBI database and validated in 35 unrelated Centre d’Etude du Polymorphisme Humain (CEPH) individuals. Of nine selected SNPs in the *ADRB2* gene (GenBank accession no. M15169; http://www.ncbi.nlm.nih.gov/entrez/viewer.fcgi?db=nuccore&id=178201), two were monomorphic or had very low minor allele frequency (rs1800888 and rs3729943), three had a success rate below 85%, the call rate cutoff used in the 35-sample validation step [rs1042711, rs1042713/Arg16Gly, and rs1042719 (Arg, arginine; Gly, glycine)], and one was out of Hardy-Weinberg equilibrium (HWE) (*p* < 0.01; rs1042721), leaving three SNPs for complete analysis [rs1042714/ Glu27Gln, rs1042717, and rs1042718 (Glu, glutamate; Gln, glutamine)]. Of seven potential SNPs in the *TNF* gene (GenBank accession no. M16441; http://www.ncbi.nlm.nih.gov/entrez/viewer.fcgi?db=nuccore&id=339739), one was monomorphic (rs3093665), and one had a success rate below 85% (rs1800630/–863), leaving five SNPs for complete analysis (rs1799964/ –1031, rs1799724/–857, rs1800629/–308, rs1800610, and rs3093664). Out of seven selected *GSTP1* (GenBank accession no. M24485; http://www.ncbi.nlm.nih.gov/entrez/viewer.fcgi?db=nuccore&id=341173) SNPs, one we excluded because of success rate below 85% (rs1871041) and included the remaining six SNPs in the study [rs762803, rs947894/ Ile105Val, rs749174, rs1799811/Ala114Val, rs1871042, and rs4891 (Ala, alanine)].

We then analyzed the included SNPs in all wheezing cases and controls (*n* = 982). We designed primers for multiplexing PCR and extension reactions by the SpectroDesigner software (Sequenom, San Diego, CA, USA) and performed PCR and extension reactions according to standard protocols (detailed information is available from the authors on request). We performed the SNP analysis by matrix-assisted laser desorption/ionization–time of flight (MALDI-TOF) mass spectrometry (Sequenom), which has an automatic quality control program that checks all genotypes with regard to consistency of genotypes, peak intensity, signal-to-noise ratio, and the like. In addition to using this program, we also manually double-checked all genotypes as an extra quality control step.

All SNPs included for analyses (Table 1) were in HWE (*p* > 0.01) and had a genotyping success rate of 93–99%.

### Statistical analyses

We evaluated deviations of observed genotype frequencies from HWE by the chi-square test. To check the haplotype inheritance of the markers we estimated the correlation (*r*^2^) and linkage disequilibrium (LD) coefficient (*D*′) using Haploview 3.2 (Barrett et al. 2005). We estimated haplotype frequencies in cases and controls combined using the expectation maximization (EM) algorithm. As an initial step, we screened main genetic effects by allelic association tests because this provides maximum power for testing in most cases even if the true genetic model is not log-additive. We obtained *p*-values for differences in allele (and haplotype) frequencies between cases and controls using the chi-square test and used a linear regression model to test for associations with mean PEF values. For the primary interaction test analyses, we used model-free (unconstrained) genotype coding with indicator (dummy) variables for heterozygote and rare homozygote genotypes to investigate the pattern of effects across different genotypes, except for SNPs where the rare homozygotes were too few, in which case we used a dominant genetic model. We then used multiple regression modeling (linear regression for PEF) to test for gene–environment interactions between genotypes and exposure to traffic NO_x_ by adding an interaction term between the genotype and exposure of interest. We performed a likelihood-ratio test between the models with and without interaction terms to test for genotype–exposure interaction. Thus, the obtained *p*-value indicates a departure from a multiplicative interaction model on the odds ratio (OR) scale and indicates whether the effect of a certain exposure is significantly altered by presence of a genotype (Table 2). For power reasons, we used a dominant coding model to assess the genotype-specific OR for wheezing or sensitization (linear regression for PEF) related to a difference between the 5th and 95th percentile of traffic NO_x_ exposure (analyzed as a continuous variable) during the first year of life (Tables 3, 4). Thus, we have used different genetic models in the different tables, which precludes direct comparison between the effect measures. In the regression model for wheezing and sensitization as outcomes we adjusted for a number of potential confounders: municipality (four categories), socioeconomic status (six categories based the parents’ occupation), heredity for allergic diseases in three categories (none, one, or both parents), maternal smoking during pregnancy and/or in infancy (yes/no), construction year of the residence (in three categories), dampness or mold in the home at birth (yes/no), and sex of the child. We also tested ethnicity in the model, but it had no confounding effect, so we did not include it in the final model. We performed a permutation test to correct for multiple testing of allelic and haplotype associations using Haploview (Barrett et al. 2005). We treated the genotypes and haplotypes as fixed, whereas we randomized the phenotypes (case or control). The proportion of 10,000 such iterations (randomized chi-square tests) that showed a stronger association than in the actual data provided the empirical *p*-value after correction for multiple tests. For correction of multiple interaction tests, we used the false discovery rate method developed by Benjamini and Hochberg (1996) to assess a *p*-value cutoff that corresponds to a nominal value of < 0.05 after correction for multiple tests.

## Results

**Figure 2:**
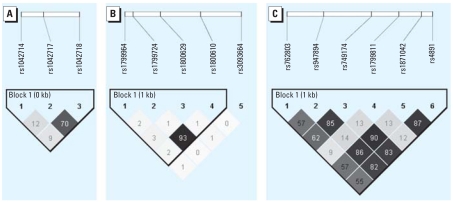
Block structure and correlation of the SNPs in the *ADRB2* gene (*A*), *TNF* gene (*B*), and *GSTP1* gene (*C*). We defined the block structure based on the LD coefficient *D*′ using Haploview (solid spine of LD) (Barrett et al. 2005). The numbers in each box correspond to the pairwise correlation coefficient *r*^2^ between respective SNPs.

We performed screening for overall main genetic effect using allelic association tests (Table 1). We observed significant associations between *GSTP1* Ala114Val and asthma [Val vs. Ala: OR = 2.1; 95% confidence interval (CI), 1.4–3.3] and between *TNF*-308 and sensitization to inhalant and/or food allergens (OR = 1.6; 95% CI, 1.1–2.2, A vs. G), with *p*-values < 0.05 after correction for multiple tests. The *GSTP1* 114Val allele also had a suggestive association with increased risk of persistent wheezing, and *TNF*-308A with asthma and persistent wheezing (*p* < 0.05 unadjusted). We saw no clear main genetic effects for *ADRB2*. Estimation of the correlation and LD between the SNPs indicated relatively high correlation in the *GSTP1* gene but generally low correlation in the *TNF* gene [Figure 2A–C; see also Supplemental Material, Figure 1A–C (http://www.ehponline.org/members/2008/11117/suppl.pdf)]. Based on the LD pattern, we defined one haplotype block in each gene [see Supplemental Material, Figure 1A–C (http://www.ehponline.org/members/2008/11117/suppl.pdf)]. We also saw the association between *TNF* and sensitization in haplo-type analyses of the four SNPs in the block (e.g., TCAC, *p* < 0.005), whereas haplotype associations in the *ADRB2* and *GSTP1* genes were not significant after correction for multiple tests (data not shown).

We next assessed gene–environment interaction effects using exposure to traffic NO_x_ during the first year of life as the environmental exposure [for main effects of traffic NO_x_ exposure on all outcomes, see Supplemental Material, Table 2 (http://www.ehponline.org/members/2008/11117/suppl.pdf)]. Interaction effects were suggestive between all tested SNPs in the *GSTP1* gene (including the coding Ile105Val and Ala114Val SNPs, *p*_nominal_ < 0.001–0.06) and exposure to traffic NO_x_ during the first year of life for sensitization at 4 years of age (Table 2). Correction for multiple tests did not change the interpretation of the results. Table 3 lists detailed results and effect estimates for Ile105Val and Ala114Val (coding SNPs) interactions. Children with a 105Val allele (Ile105Val or Val105Val genotype) had almost a 2.5-fold increased risk of sensitization (≥ 0.35 kU_A_/L) when exposed to elevated levels of traffic NO_x_ during the first year of life (for a difference between the 5th and the 95th percentile of NO_x_ exposure, which corresponds to 44.1 μg/m^3^ difference; OR = 2.4; 95% CI, 1.0–5.3), whereas we observed no increased risk in children homozygous for Ile/Ile. For comparison, Table 3 also presents the overall main effects (i.e., disregarding possible interaction) for these *GSTP1* genotypes and for exposure to traffic NO_x_ during the first year of life, respectively. Using a higher IgE cutoff level for sensitization (≥ 3.5 kU_A_/L), we found a similar association to traffic NO_x_ in the Ile105Val/Val105Val group (OR = 2.5; 95% CI, 0.9–6.8). Interaction with respect to PEF values was also suggestive for two *GSTP1* SNPs, including Ile105Val (Table 2). Children carrying the Ile105Val or Val105Val genotype had the largest decrease in their PEF values associated with the difference between the 5th and 95th percentile of exposure to traffic NO_x_ (on average, −7.2 L/min; 95% CI, −15.0, 0.5; compared with Ile/Ile subjects, −1.7 L/min; 95% CI, −11.0, 7.7). However, the interaction *p*-value for PEF analyses was not significant after correction for multiple tests. The other coding *GSTP1* SNP, Ala114Val, showed an interaction pattern similar to that for Ile105Val for sensitization (Table 3), and we saw similar results for the other *GSTP1* SNPs, with heterozygous/rare homozygous genotype carriers showing increased risk for sensitization but the common homozygous individuals showing no significant risk (data not shown). In general, the increased risk seemed to be largely driven by the heterozygote individuals (e.g., for sensitization in Ile105Val heterozygotes, OR = 3.9; 95% CI, 1.4–10.8). However, the number of individuals in such subgroup analyses is very small (especially for the rare homozygote genotype), and this aspect of our results should be interpreted with caution.

We observed no significant interactions between variants in the *TNF* and *ADRB2* genes and exposure to traffic NO_x_ with regard to respiratory symptoms, PEF, or sensitization. However, *TNF* and *GSTP1* SNPs have been suggested to have joint effects with respect to air pollution exposures (Li et al. 2006), and we assessed whether the gene–environment effect between NO_x_ exposure and *GSTP1* Ile105Val on sensitization was further modified by the *TNF* G-308A SNP. The *GSTP1* Ile105Val–air pollution interaction with respect to sensitization was much stronger in children with the *TNF*-308 risk genotype (GA/AA) than in children with the nonrisk genotype (GG) (*p* < 0.001 vs. *p* = 0.08, respectively) (Table 4). We observed a similar trend to stronger interaction in the −308 GA/AA group with respect to PEF values and, to some extent, persistent wheeze, although it was not significant. Notably, children with the *GSTP1* Ile105Val/Val105Val and *TNF* GA/AA genotypes exposed to high levels of NO_x_ not only had the highest risk of sensitization but also showed a substantial decrease in their PEF values (on average, −27.8 L/min compared with those exposed to low levels).

As a sensitivity analysis, we also performed association and interaction tests using sensitization and PEF as outcomes in the representative subcohort of 635 children. The results and interpretations were very similar to from analyses including all children (both wheezing cases and controls), which supports the general significance of the findings (data for subcohort only not shown).

## Discussion

We present results that support a role of interactions between a natural antioxidant enzyme, *GSTP1*, and long-term exposure to ambient air pollution from traffic with respect to sensitization to common allergens in children. *TNF* and *ADRB2* polymorphisms did not seem to directly influence the effect of air pollution from traffic on the development of asthmatic symptoms and sensitization, but in combination with *GSTP1*, *TNF* substantially modified the effect of traffic-related exposure.

The GSTP1 enzyme is of particular interest in relation to the respiratory system because it has been reported to provide more than 90% of the GST activity in the lung (Fryer et al. 1986). The GSTP1 enzymatic activity has been shown to differ between isoenzymes with Ile or Val in amino acid position 105, with Val105 enzymes having a higher catalytic capacity for polycyclic aromatic hydrocarbons, which represent a widespread class of environmental pollutants, but a lower conjugation capacity for, among others, 1-chloro-2,4-dini-trobenzene (Sundberg et al. 1998; Watson et al. 1998). The active GSTP1 enzyme in the cytosol consists of two subunits that form a dimer (Reinemer et al. 1991). GSTP1 also physically interacts with other molecules and contributes to other biologic pathways, such as c-Jun N-terminal kinase (JNK), a protein kinase involved in transcription of cytokines, immunoglobulins (e.g., IgE), and inflammatory enzymes, and 1-cystein peroxiredoxin (1-Cys Prx), which catalyzes reduction of peroxides (Bennett et al. 2001; Ralat et al. 2006). Dimerization of GSTP1 subunits generates a catalytically active enzyme, whereas heterodimerization of GSTP1 with either JNK or 1-Cys Prx is a mechanism by which the GSTP1 enzyme exerts additional regulatory and antioxidative effects.

Conflicting results on allergy and asthma have been presented for individuals homozygous for the Val105 variant. Although some studies have reported a protective effect for childhood respiratory illness (Carroll et al. 2005; Fryer et al. 2000; Gilliland et al. 2002c), others show an association with slower lung function growth, especially if the child suffers from asthma (Gilliland et al. 2002a). Further, two studies were unable to show any effect of Val105 homozygosity on asthma or atopy (Brasch-Andersen et al. 2004; Nickel et al. 2005). In the present study, we found no main effect of Ile105Val polymorphism for the tested outcomes. Instead, another coding variant, Ala114Val, was associated with an increased risk (Val) of asthma at 4 years of age. However, no association was found between this polymorphism and asthma in two German pediatric populations (Nickel et al. 2005). Like many other studies, we based our asthma diagnosis on parentally reported doctor’s diagnosis, which we did not confirm by a study physician or any objective measurements, although the latter is difficult for this age group. The lack of a detailed, objective asthma definition is, of course, a limitation in our study and may explain some discrepancies. Another possible explanation for the divergent findings is that the genetic effect manifests clearly only in interaction with environmental exposures, which our results suggest.

Previous interaction studies support the role of *GSTP1* variants modifying the effect of exposure to air pollutants and tobacco smoke with respect to asthma and allergy. In an experimental study, 19 ragweed-sensitized adults were challenged intranasally with allergen alone and with allergen plus diesel exhaust particles (Gilliland et al. 2004). Patients homozygous for the Ile105 variant showed the largest histamine release and increase in nasal IgE responses after challenge, and the same Ile105 homozygote subjects also had the largest IgE response after challenge with second-hand tobacco smoke in a follow-up study (Gilliland et al. 2006). A study on 61 asthmatic and 95 nonasthmatic school children showed that the Ile105Ile genotype conferred an increased asthma risk only in districts with high air pollution (defined by high NO_x_ and SO_2_ levels) (Lee et al. 2004). The Val105 allele frequencies differ somewhat between these studies, 16% and 22%, respectively, compared with 33% in our material (no difference between boys and girls). For HapMap CEPH samples (from Utah residents with northern and western Europe ancestry), it is 39% (Altshuler et al. 2005).

High correlation between the *GSTP1* SNPs implies that we cannot directly identify the SNP responsible for the observed interaction pattern. Most other studies have focused on the functional Ile105Val rather than Ala114Val, because of the low allele frequency and unknown functional effects of the latter. In the interactions seen between Ile105Val and traffic NO_x_ with respect to sensitization, and the weaker effect but with a similar pattern with respect to PEF values, carriers of the 105Val allele (Ile/Val or Val/Val individuals) consistently showed the highest risk estimates in relation to air pollution levels. These results contrast with previous studies, where Ile105 homozygotes seem to have the greatest IgE response after air pollution or tobacco smoke challenge (Gilliland et al. 2004, 2006). Other studies have, however, shown that asthmatic children with two Val105 alleles seem more likely to experience respiratory symptoms in response to acute exposure to ozone, rather than Ile105 homozygotes (Romieu et al. 2006). A recently published study also showed that children with *GSTP1* Val/Val genotype and a high activity for EPHX1 (microsomal epoxide hydrolase, also part of the antioxidative system) were at the highest asthma risk, especially if they lived < 75 m from a major road (Salam et al. 2007).

Whether there are different antioxidant mechanisms of GSTP1 in response to short- or long-term exposure to specific air pollutants remains to be investigated, as well as possible differences between effects seen in allergic or asthmatic patients compared with the risk of developing a disease in previously healthy individuals. Timing of exposure may also be crucial, and, in contrast to the studies referred to above, we present data on exposure very early in life, before onset of any symptom. By using exposure during the first year of life only, we minimize possible reverse causality induced by avoidance behavior due to the child’s disease. At this age, the air pollution levels at the home address are also highly relevant as exposure estimates, because only 1% of the children in the study started day care before 12 months of age. Individual exposure estimates for the different life years were highly correlated, limiting the possibility of estimating effects related to specific time windows of exposure (Nordling et al. 2008). However, previous results support the importance of environmental exposure during the first year of life, especially for sensitization (Lannero et al. 2008).

Although TNF seems to be a key player for the inflammatory response following exposure to air pollutants, we found no evidence of effect modification by variants in this gene on the outcomes tested in the initial analyses. However, in combination with *GSTP1*, we found a strong effect modification of *TNF* G-308A, in that the risk of sensitization and loss of lung function following NO_x_ exposure was greatest in children with *GSTP1* Ile105Val/ Val105Val and *TNF* GA/AA genotypes. The −308A variant has been of particular interest because of enhanced *in vitro* transcription and increased TNF levels in white blood cells (Wilson et al. 1997). Inconsistent findings have been reported on the overall influence of −308A on susceptibility to asthma and allergy, although a recent meta-analysis concluded that the A allele confers a significant risk of asthma (Gao et al. 2006). The same allele was also associated with sensitization in the main effect analyses in our study. Importantly, the G-308A polymorphism has been shown to modify the effect of both ozone and secondhand tobacco smoke on respiratory illness, although the direction of the modification differs between the studies (both G and A alleles associated with highest risk following exposure) (Li et al. 2006; Wenten et al. 2005; Wu et al. 2007; Yang et al. 2005). Particularly, the protective effect of *TNF* GG genotype (alternatively, the risk effect of *TNF* GA/AA) seem to be related to asthma risk predominantly in subjects from nonsmoking families or children in low-ozone-exposure communities (Li et al. 2006; Wu et al. 2007). In the study by Li et al. (2006), the protective effect of *TNF* GG was thus reduced in high-ozone communities, especially in *GSTP1* Ile105Ile individuals, which suggests that the effect of *TNF* genotype is dependent on the oxidative defense capacity. Our findings replicate to some extent the results of Li et al. (2006) and support the dependency between *GSTP1* and *TNF* in relation to air pollution exposures. The *TNF* risk genotype (GA/AA) seems to potentiate the risk associated with exposure, and we speculate that increased TNF levels may in part explain these observations, based on previous results on increased expression associated with the A allele (Wilson et al. 1997). However, the combined functional effect of *GSTP1* and *TNF* polymorphisms in relation to air pollution exposures remains to be elucidated, as well as the role of GSTP1 in relation to JNK or 1-Cys Prx as discussed above.

Polymorphisms in the *ADRB2* gene (especially Gly16Arg) have been reported to affect not only the long-term response to ADRB2 agonists but also the risk of asthma in relation to active and passive smoking, and we specifically selected *ADRB2* in this study based on previous interaction effects (Israel et al. 2004; Wang et al. 2001; Zhang et al. 2007). Unfortunately, we were unable to study the effect of Gly16Arg because of genotyping problems with the technology used, and we found no interaction for the three remaining tested polymorphisms, after exclusion for monomorphic status or deviation from HWE. However, information from the HapMap project shows strong LD between Gly16Arg and the Gly27Gln variant we studied (*D*′ = 0.94, *r*^2^ = 0.38) (Altshuler et al. 2005). To our knowledge, no other study to date has been published on *ADRB2* interactions with air pollutants other than smoking.

Different gene–environment effects in different settings may occur for several reasons. The pattern of exposure in observational studies can differ substantially between countries or areas, and the genotype frequencies also show large global differences (Altshuler et al. 2005). Differences in genotype frequencies have also been observed in relatively homogenous populations such as in Sweden (Hannelius et al. 2005). The BAMSE study population was recruited from the Stockholm area, and only 16% of the children have one or two parents born outside Scandinavia. We also included ethnicity in the analyses as a potential confounder, but it had no such effect on the interaction analyses. This should rule out any major influence of population stratification. Insufficient power is also a major concern in studies on gene–environment interactions, because the number of individuals with each combination of a specific genetic variant and environmental exposure will be low. A continuous-exposure variable such as NO_x_ in this study increases the power to detect interactions, but the number of individuals with different genotypes who are cases or controls is still limited. Therefore, we cannot rule out the possibility that we obtained false-negative results because of low power. In the whole BAMSE cohort, NO_x_ exposure was associated with sensitization, especially to pollen (e.g., for traffic NO_x_, OR = 1.67; 95% CI, 1.10–2.53) (Nordling et al. 2008). In this subsample of the BAMSE study, it would have been interesting to look at interaction with respect to specific allergens such as pollen, but because of insufficient power for subgroup analyses, we analyzed inhalant and food allergens jointly. In general, both the main genetic effects (e.g., *TNF*-308 SNP) and interaction effects (e.g., *GSTP1* Ile105Val–NO_x_) were somewhat stronger for inhalant allergens than for food allergens, but the differences were small, and the directions of association were similar (data not shown).

There are many practical issues to consider when attempting examining lung function in a large group of 4-year-olds. To ensure good-quality measurements, we used a reproducibility criterion in addition to the test leader’s opinion of the child’s effort. The usefulness of the PEF measurement could be criticized, because it may not correlate well with other measures used in the diagnostic procedure of asthma. However, we compared the effect on airflow between groups of children and did not attempt to use the PEF measurements as a tool for diagnosis or diagnostic criterion. Further, in a previous publication on the same cohort, we were able to show that PEF is significantly reduced in groups of children with asthma compared with healthy children (Hallberg et al. 2006).

The possibility of false-positive findings also needs to be considered, and we have addressed the multiple testing problems with false discovery rate analyses, which suggest that the observed *GSTP1*–NO_x_ interaction is likely not to be a false positive. There is, however, a lack of functional data from experimental studies to directly support our findings of gene–environment interactions. Because we found a high correlation between exposure to traffic NO_x_ and traffic PM_10_ in this data set, we cannot disentangle effects between NO_x_ and PM_10_ or, for that matter, other traffic-related pollutants such as ultrafine particles. Thus, exposure to source-specific NO_x_ in this study should rather be viewed as a proxy for traffic-related air pollutants.

In conclusion, our data indicate gene–environment interaction effects on the development of allergic diseases in childhood. Specifically, variants in the *GSTP1* and *TNF* genes seemed to modify the effect of early long-term exposure to ambient air pollution from traffic with respect to sensitization to common allergens in children. These results support the role of genes controlling the antioxidative system and inflammatory response in the pathogenesis of allergy and asthma.

## Figures and Tables

**Table 1:** Association of asthma, wheezing, sensitization, and mean PEF values in 4-year-old children from a birth cohort in Stockholm for SNPs in the *ADRB2*, *TNF*, and *GSTP1* genes.

				Allelic associations (*p*-value^a^)					
Gene symbol	SNP (major/minor allele)	Localization and coding change	MAF (*n* = 982)	Asthma (*n* = 136 cases)	Transient wheezing (*n* = 155 cases)	Late-onset wheezing (*n* = 131 cases)	Persistent wheezing (*n* = 211 cases)	Sensitization^b^ (*n* = 266 cases)	PEF^c^ (*n* = 860)
*ADRB2*	rs1042714 (C/G)	Exon 1, Glu27Gln	0.41	0.68	0.54	0.94	0.53	0.09	0.92
	rs1042717 (G/A)	Exon 1	0.19	0.41	0.11	0.32	0.18	0.80	0.92
	rs1042718 (C/A)	Exon 1	0.15	0.49	0.15	0.33	0.24	0.63	0.76
TNF	rs1799964 (T/C)	−1031	0.21	0.78	0.33	0.41	0.51	0.40	0.58
	rs1799724 (C/T)	−857	0.09	0.67	0.009^d^	0.24	0.91	0.95	0.78
	rs1800629 (G/A)	−308	0.16	0.03^d^	0.94	0.51	0.03^d^	<0.005^e^	0.26
	rs1800610 (C/T)	Intron 1	0.09	0.96	0.03^d^	0.26	0.87	0.99	0.87
	rs3093664 (A/G)	Intron 3	0.08	0.47	0.32	0.75	0.93	0.75	0.87
*GSTP1*	rs762803 (C/A)	Intron 4	0.41	0.26	0.16	0.39	0.26	0.36	0.81
	rs1695 (A/G) (rs947894)	Exon 5, Ile105Val	0.33	0.97	0.57	0.38	0.90	0.93	0.48
	rs749174 (C/T)	Intron 5	0.34	0.36	0.55	0.94	0.30	0.83	0.70
	rs1138272 (C/T) (rs1799811)	Exon 6, Ala114Val	0.09	<0.001^e^	0.44	0.17	0.03^d^	0.35	0.75
	rs1871042 (C/T)	Intron 6	0.33	0.47	0.78	0.45	0.19	0.87	0.78
	rs4891 (T/C)	Exon 7	0.31	0.91	0.61	0.64	0.51	0.97	0.17

MAF, minor allele frequency.

a We obtained *p*-values from the chi-square test. Nonwheezing children (*n* = 485) served as controls for asthma and the wheezing outcomes.

b Sensitization to inhalant and/or food allergens (IgE ≥ 0.35 kU_A_/L). We analyzed all available sensitized children, both wheezing cases and controls (nonwheeze, nonsensitized children as controls, *n* = 362).

c *p*-Values for difference in PEF mean values for the rare allele versus common allele [linear regression model with model-free coding of genotypes (indicator variables)]. We included all genotyped children, both wheezing cases and controls, and we adjusted the analyses for age, sex, and height (860 of the 982 genotyped children had acceptable PEF values).

d *p*-Value > 0.05 after 10,000 permutations.

e *p*-Value 0.005 (asthma) and 0.01 (sensitization) after 10,000 permutations.

**Table 2:** Interaction between SNPs in each gene and exposure to traffic NO_x_ during the first year of life with regard to respiratory outcomes, sensitization, and mean PEF at 4 years of age in children from a birth cohort in Stockholm.

			Nominal *p*-values for interaction^a^					
Gene symbol	SNP	Localization and coding change	Asthma (*n* = 136 cases)	Transient wheezing (*n* = 155 cases)	Late-onset wheezing (*n* = 131 cases)	Persistent wheezing (*n* = 211 cases)	Sensitization^b^ (*n* = 266 cases)	PEF^c^ (*n* = 860)
*ADRB2*	rs1042714 (C/G)	Exon 1, Glu27Gln	0.56	0.39	0.28	0.58	0.10	0.41
	rs1042717 (G/A)	Exon 1	0.51	0.73	0.22	0.94	0.20	0.35
	rs1042718 (C/A)	Exon 1	0.06	0.56	0.39	0.59	0.38	0.22
*TNF*	rs1799964 (T/C)	−1031	0.73	0.22	0.21	0.53	0.80	0.50
	rs1799724 (C/T)	−857	0.74	0.61	0.13	0.54	0.12	0.46
	rs1800629 (G/A)	−308	0.41	0.47	0.09	0.63	0.57	0.80
	rs1800610 (C/T)	Intron 1	0.74	0.72	0.14	0.69	0.22	0.43
	rs3093664 (A/G)	Intron 3	0.8	0.05	0.33	0.15	0.63	0.33
*GSTP1*	rs762803 (C/A)	Intron 4	0.26	0.44	0.79	0.29	< 0.001^a^	0.29
	rs1695 (A/G) (rs947894)	Exon 5, Ile105Val	0.22	0.32	0.73	0.6	0.001^a^	0.04^a^
	rs749174 (C/T)	Intron 5	0.79	0.37	0.87	0.92	0.01^a^	0.24
	rs1138272 (C/T) (rs1799811)	Exon 6, Ala114Val	0.93	0.69	0.89	0.44	0.06	0.64
	rs1871042 (C/T)	Intron 6	0.24	0.46	0.76	0.69	0.004^a^	0.12
	rs4891 (T/C)	Exon 7	0.25	0.6	0.65	0.34	0.003^a^	0.006^a^

We adjusted all analyses for municipality, socioeconomic status, heredity, mother’s smoking during pregnancy, year the house was built, dampness or mold in the home (at birth), and sex of the child, except for the PEF analyses, which we adjusted for age, sex, height, and municipality. We coded each SNP model free (indicator variables), except for rs1799811, rs1799724, rs1800610, and rs3093664 (coded 0, 1 because of few rare homozygous individuals).

a We obtained *p*-values for departure from a multiplicative interaction model by likelihood-ratio tests between the models with and without interaction terms. Corrected *p*-value cutoffs after correction for multiple tests: 0.01 (corrected for tests on 14 SNPs and sensitization as outcome), < 0.006 (corrected for tests on 14 SNPs and PEF as outcome), 0.01 (corrected for sensitization and PEF), and < 0.0001 (corrected for all outcomes in the table).

b Sensitization to inhalant and/or food allergens (IgE ≥ 0.35 kUA/L) in all children, both wheezing cases and controls (nonwheeze, nonsensitized children as controls, *n* = 362).

c We included all genotyped children, both wheezing cases and controls, in the PEF analyses (860 of the 982 genotyped children had acceptable PEF values).

**Table 3:** Overall main effects of *GSTP1* (Ile105Val and Ala114Val) genotypes and NO_x_ exposure: effect modification of NO_x_ exposure, by *GSTP1* genotypes, on sensitization at 4 years of age in children from a birth cohort in Stockholm.

	Sensitization^a^	
Effect	Cases/Controls (no.)	OR^b^ (95% CI)
Main effects of *GSTP1* and NO_x_		
rs947894, Ile105Val		
Ile/Ile	113/161	1.0 (referent)
Ile/Val, Val/Val	149/195	1.0 (0.7–1.4)
rs1799811, Ala114Val		
Ala/Ala	211/296	1.0 (referent)
Ala/Val, Val/Val	41/47	1.1 (0.7–1.9)
NO_x_ effect	266/362	1.4 (0.7–2.8)
Effect of NO_x_ exposure by *GSTP1* genotype, effect modification		
By rs947894, Ile105Val		
Ile/Ile	113/161	0.5 (0.2–1.5)
Ile/Val, Val/Val	149/195	2.4 (1.0–5.3)
By rs1799811, Ala114Val		
Ala/Ala	211/296	1.2 (0.5–2.9)
Ala/Val, Val/Val	41/47	4.2 (1.0–18.7)

a Sensitization to inhalant and/or food allergens in both wheezing cases and controls at 4 years of age using a cutoff level of IgE ≥ 0.35 kU_A_/L.

b We calculated ORs for traffic NO_x_ exposure during the first year of life for the difference between the 5th and 95th percentile range of exposure in the cohort, which corresponds to 44 μg/m3 (mean for controls, 22.3 μg/m3). We calculated OR for GSTP1 genotypes using standard procedures. We adjusted all analyses for municipality, socioeconomic status, heredity, mother’s smoking during pregnancy, year the house was built, dampness or mold in the home (at birth), and sex of the child. For main effects of *GSTP1* genotypes and traffic NO_x_ exposure, we used a model without gene–environment interaction.

**Table 4:** Effect modification of traffic NO_x_ effects on different outcomes at 4 years of age by *GSTP1* Ile105Val and *TNF* G-308A genotypes.

		Sensitization^a^		Asthma		Persistent wheezing			
*GSTP1* Ile105Val	*TNF* –308	Ca/Co (no.)	OR^c^ (95% CI)	Ca/Co (no.)	OR^c^ (95% CI)	Ca/Co (no.)	OR^c^ (95% CI)	PEF^b^ (no.)	Difference (L/min)
Ile/Ile	GG	72/115	0.6 (0.2–2.0)	31/144	0.9 (0.1–5.8)	52/144	3.3 (0.8–13.2)	245	1.6 (−9.3, 12.5)
Ile/Val, Val/Val	GG	87/142	1.7 (0.7–4.1)	47/179	2.9 (1.0–8.2)	69/173	3.2 (1.3–7.7)	314	−2.4 (−10.9, 6.1)
*p*-Value interaction^d^			0.08		0.16		0.95		0.45
Ile/Ile	GA/AA	33/37	0.5 (0.1–4.0)	21/59	0.8 (0.1–5.8)	29/54	0.8 (0.1–1.6)	102	−7.4 (−27.8, 13.1)
Ile/Val, Val/Val	GA/AA	52/42	22.0 (1.6–298)	26/69	3.2 (0.5–21.7)	39/68	2.3 (0.4–12.9)	125	−27.8 (−46.8, −8.9)
*p*-Value interaction^d^			<0.001		0.15		0.20		0.05
*p*-Value overall interaction (three-way)^e^			0.001		0.40		0.65		0.32

Abbreviations: Ca, cases; Co, controls.

a Sensitization to inhalant and/or food allergens, IgE ≥ 0.35 kU_A_/L in both wheezing cases and controls (nonwheeze, nonsensitized children).

b We included all genotyped children (both wheezing cases and controls) in the PEF analyses.

c We calculated ORs (and PEF difference) for traffic NO_x_ exposure during the first year of life for the difference between the 5th and 95th percentile range of exposure in the cohort, which corresponds to 44 μg/m3 (mean for nonwheezing controls, 22.3 μg/m3). We adjusted all analyses for municipality, socioeconomic status, heredity, mother’s smoking during pregnancy, year the house was built, dampness or mold in the home (at birth), and sex of the child, except for the PEF analyses, which we adjusted for age, sex, height, and municipality.

d *p*-Value for interaction between *GSTP1* genotypes and NO_x_ in each subgroup of *TNF*-308 GG and GA/AA individuals, respectively.

e *p*-Value for overall interaction between *GSTP1* genotypes, NO_x_, and *TNF* genotypes (three-way).
